# Asynchronous presentation of global and local information reveals effects of attention on brain electrical activity specific to each level

**DOI:** 10.3389/fpsyg.2014.01570

**Published:** 2015-01-13

**Authors:** Jorge Iglesias-Fuster, Yusniel Santos-Rodríguez, Nelson Trujillo-Barreto, Mitchell J. Valdés-Sosa

**Affiliations:** ^1^Cognitive Neurosciences, Cuban Center for NeuroscienceHavana, Cuba; ^2^Neuroinformatics, Cuban Center for NeuroscienceHavana, Cuba

**Keywords:** compound figure, ERP, global, local, attention

## Abstract

The neural basis of selective attention within hierarchically organized Navon figures has been extensively studied with event related potentials (ERPs), by contrasting responses obtained when attending the global and the local echelons. The findings are inherently ambiguous because both levels are always presented together. Thus, only a mixture of the brain responses to two levels can be observed. Here, we use a method that allows unveiling of global and local letters at distinct times, enabling estimation of separate ERPs related to each level. Two interspersed oddball streams were presented, each using letters from one level and comprised of frequent distracters and rare targets. Previous work and our Experiment 1 show that it is difficult to divide attention between two such streams of stimuli. ERP recording in Experiment 2 evinced an early selection negativity (SN, with latencies to the 50% area of about 266 ms for global distracters and 276 ms for local distracters) that was larger for attended relative to unattended distracters. The SN was larger over right posterior occipito-temporal derivations for global stimuli and over left posterior occipito-temporal derivations for local stimuli (although the latter was less strongly lateralized). A discrimination negativity (DN, accompanied by a P3b) was larger for attended targets relative to attended distracters, with latencies to the 50% area of about 316 ms for global stimuli and 301 ms for local stimuli, which presented a similar distribution for both levels over left temporo-parietal electrodes. The two negativities apparently index successive stages in the processing of a selected level within a compound figure. By resolving the ambiguity of traditional designs, our method allowed us to observe the effects of attention for each hierarchical level on its own.

## Introduction

**Figure 1:**
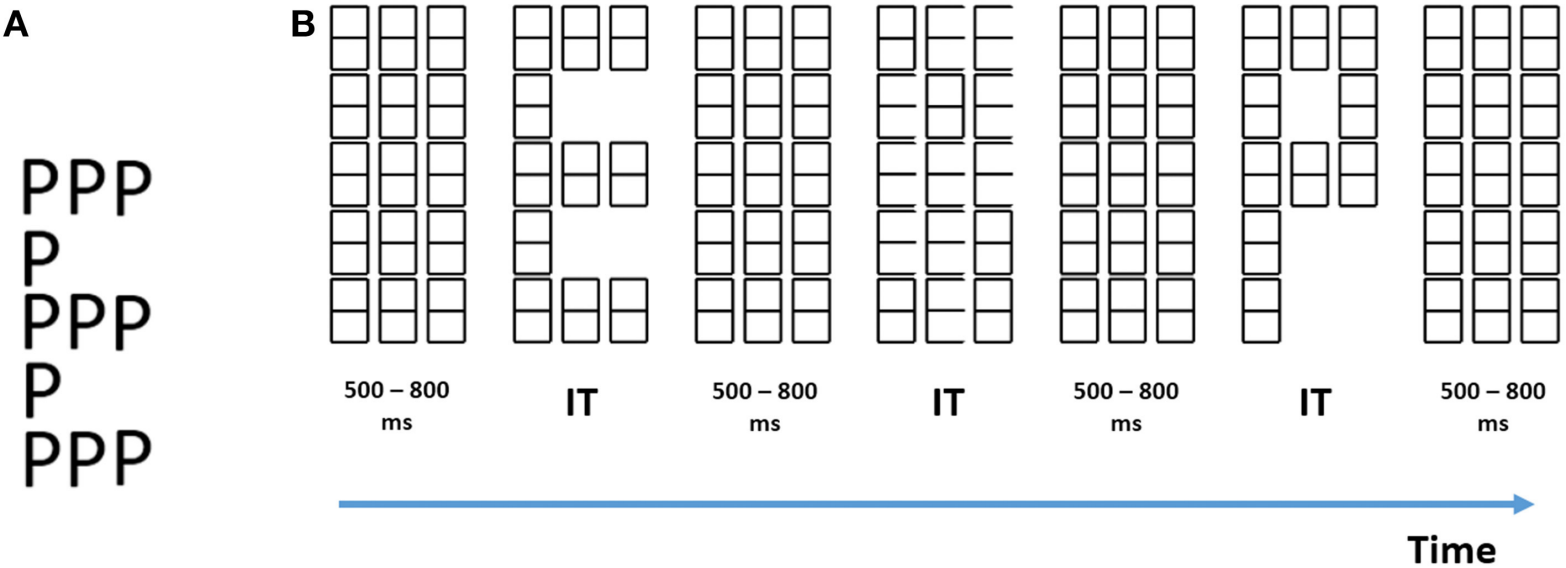
**(A)** Example of a traditional compound letter described by Navon (1977). **(B)** Example of our stimuli. Successive elements from left to right are: the baseline (mask) matrix, a global “E,” another mask, a local “E,” another mask, a global “P” and finally another mask. IT, individually titrated stimulus durations.

Visual scenes can be perceived at different hierarchical levels, which go from the most global tier down to the finest details (e.g., crowd-person-face-eyes-eyelashes). This aspect of scene organization has been artificially mimicked in the laboratory by using compound letters (i.e., a global letter made out of local letters, see Figure 1A). These letters are rapidly recognized when attention is focused on only one echelon, but this process is slowed down when attention must be divided between levels (Navon, 1977). Despite continued interest in this type of stimuli (for a review see Kimchi, 2013), the neural mechanisms used to allocate attention across levels are not clearly understood. How early in visual processing does attentional selection of hierarchical levels take place? Does the selection of level take place before letter identity is established, or does it come afterwards? Are distinct neural systems used to process the different levels and does their activation in time differ?

Similar issues have been previously addressed with visual event related potentials (ERPs) for spatial-based (Anllo-Vento and Hillyard, 1996; Clark and Hillyard, 1996; Hillyard and Anllo-Vento, 1998), feature-based (Hopf et al., 2004; Nobre et al., 2006; Andersen et al., 2008), and object-based attention (Valdes-Sosa et al., 1998; Martinez et al., 2007), using elegant methods painstakingly developed over decades (Hillyard et al., 1973; Hillyard and Picton, 1979; Kappenman and Luck, 2012). Some of these techniques have also been applied to study global/local processing, but several obstacles preclude application of the more powerful methods including what is known as the Hillyard sustained attention paradigm (Luck and Kappenman, 2012). We briefly summarize previous work on the ERP effects of attention to hierarchical stimuli. We later analyze the obstacles mentioned above and then propose a line of attack to overcome these limitations.

To establish which ERP components are modulated by attention, we need to compare signals elicited by exactly the same stimuli but obtained in two different conditions: once when attended and another when ignored (Hillyard et al., 1973; Hillyard and Picton, 1979; Kappenman and Luck, 2012). This segregates attentional effects from those due to physical changes in the stimuli. In the case of global/local processing, this has been achieved by presenting the same set of compound letters twice and consecutively directing attention toward each level (Han et al., 1997, 1999, 2001; Volberg and Hübner, 2004; Machinskaya et al., 2010). Most studies employing this strategy have used a slow event-related design (inter-trial intervals in the order of seconds), that has the advantage of reducing overlap of responses from successive trials, but at the cost of reducing the observer's processing load which makes attentional selection less necessary (Lavie and Tsal, 1994; Lavie, 1995).

The overall conclusion from this work (see Flevaris et al., 2014) is that ERPs are modulated when attention switches between different hierarchical levels, especially in the N2 range (~180–300 ms). In some studies larger effects were found for the attend- local condition over the left hemisphere and for the attend–global condition over the right hemisphere (Heinze and Münte, 1993; Schatz and Erlandson, 2003; Volberg and Hübner, 2004; Flevaris et al., 2011), although in other reports this asymmetry was absent for one level (Jiang and Han, 2005). However, real consensus is lacking on the latency, polarity, and scalp topography of these effects. These discrepancies could be due to differences in the stimuli, experimental design, and in the strategies deployed by subjects across studies, as well as the methodological obstacles in applying ERPs to global/local attentional selection examined below.

These methodological obstacles are part and parcel of the experimental design used in previous work and outlined above. Firstly, although we need to examine the effects of attention on the processing of global and of local information separately, this is not possible with the designs used up to now. If one could completely shut down processing of the supposedly unattended level, then ERPs could reflect neural activity associated with a single level (the attended one). However, there is ample psychophysical evidence that this is not possible with traditional compound letters for which local and global information are always presented at the same time (see Figure 1A). For example, identification of letters at an attended level is slower when they are incongruent with the letters at the unattended level (Navon, 1977) compared to when they are congruent. Furthermore, conjunction errors (i.e., reporting letter identity from the unattended level) are frequent when stimulus presentation times are short (Hübner and Volberg, 2005). This means that ERPs elicited by traditional compound figures inevitably contain activity triggered by processing at both levels. Any change due to attention could be due to reduced activity for processes related to one level and/or increased activity to processes related to the other. Moreover, it is impossible to ascertain the polarity of the attentional ERP effect. The results are thus inherently ambiguous, and do not allow us to examine the effects of attention on global and on local processing each on its own (see the discussion of this recurrent problem in ERP research by Kappenman and Luck, 2012).

Secondly, selective attention is not fully manifested when the processing load (Lavie and Tsal, 1994; Lavie, 1995) of the task is low (i.e., at slow stimulus presentation rates). Thus, previous work may have underestimated the magnitude of global/local selection effects on ERPs. The early ERP literature reached the conclusion that fast stimulation rates were necessary to evince strong effects of auditory and visuo-spatial attention (Hillyard et al., 1973; Hillyard and Picton, 1979). However, note that fast stimulation rates elicit a large degree of response overlap between successive trials (Woldorff, 1993), which would further complicate interpretation of ERP results. This probably has discouraged use of fast stimulation rates in global/local research.

Both these obstacles need to be overcome in order to use the Hillyard sustained attention paradigm, which has the advantage of controlling for important confounding factors distinct from attention (Kappenman and Luck, 2012). In this paradigm, attention is first focussed for long blocks of trials on one of two sensory channels, and then is switched to the other. These channels can be the two ears, two locations in visual space, or two fingers, among many other variants. In each channel, stimuli are presented at fast rates in an oddball sequence (infrequent target stimuli interspersed among frequent distracter stimuli, with response required only for the former). Random inter-stimulus intervals are used. The high processing load (usually aided by difficult target/distracter discrimination) forces the subject to effectively focus attention on only one channel, thus excluding the other from further processing. The random jitter of the onsets has the effect of “smearing” contributions of all other stimuli to the average ERP that is time-locked to a specific stimulus. Thus, jitter acts as a low-pass filter (Woldorff, 1993). This enables comparison of the ERPs specifically related to stimuli from each channel when they are attended and when they are ignored.

The Hillyard paradigm has been used in numerous studies of selective attention to visual (Anllo-Vento and Hillyard, 1996; Clark and Hillyard, 1996; Luck et al., 1997; Hillyard and Anllo-Vento, 1998), auditory (Hillyard et al., 1971, 1973; Picton et al., 1971; Schwent and Hillyard, 1975; Hink and Hillyard, 1976) and somatosensory channels (Desmedt and Robertson, 1977; Desmedt et al., 1983; Michie et al., 1987). Interestingly, the Go/No go design of the Hillyard paradigm allows ERPs for distracters to be uncontaminated by components related to response selection and organization. Thus, difference waveforms obtained by subtracting responses to distracters when unattended from the corresponding responses when attended yield a fairly pure measure of neural effects due to perceptual attentional selection. Selection for visual spatial locations (Hillyard and Anllo-Vento, 1998), or competing superimposed surfaces (Valdes-Sosa et al., 1998) modulates the early and exogenously driven P1 and N1 (with probable sources in visual extra-striate cortex), but not the C1 which originates in V1 (e.g., Ding et al., 2014).

In contrast, selection for several visual features (including color, spatial frequency, and motion direction), increases the amplitude of temporal-occipital selection negativities, or SNs, that overlap exogenous components, but seem to be endogenously driven (Hillyard and Anllo-Vento, 1998). On the other hand, difference waveforms obtained by subtracting responses to distracters from the responses to oddball targets within an attended channel can index post-perceptual neural processes, usually in the form of a prominent N2/P3 complex. This can only occur after letter identity has been established (i.e., after post-perceptual processes are initiated). Although the topography of the P3 to oddball targets is invariant to stimulus characteristics (including sensory modality), N2 scalp distribution is modality-dependent and varies even with the attended intra-modality category or feature (Harter and Guido, 1980; Harter and Aine, 1984; O'Donnell et al., 1997). We will dub this component here as the discrimination negativity (DN).

Given the advantages of the Hillyard paradigm for studying the neural basis of selective attention, it seems important to modify compound stimuli to allow its application. This may seem impossible because apparently global letters need the local letters to exist (see Figure 1A). In these stimuli the onset of local and global information are always confounded, and their responses cannot be separated (the same situation would exist if stimuli to two ears or two visual hemifields were always presented in synchrony). However, separate global and local channels have been posited to exist, possibly mediated each by a distinctive visual spatial frequency band: low spatial frequencies (LSF) carrying the global level and high spatial frequencies (HSF) carrying the local level (Shulman et al., 1986; Shulman and Wilson, 1987; Robertson, 1996; Flevaris et al., 2011). If this were so, then we need to dissociate the onset times of global/local aspects of hierarchal figures, in order to study each channel separately.

We have achieved this separation by what we call by ***level specific letter presentation*** (Lopez et al., 2002; Valdes-Sosa et al., 2014a,b), a procedure that allows us to display at any given time only one informative level within a compound figure. The procedure consists of presenting a matrix of place-holding figures (that also functions as a mask). Either global or local letters can be selectively unveiled by erasure of selected line segments (see Figure 1B), while a non-informative distracter figure is presented at the other level. This means that it is possible to trigger ERPs linked to the onset of only one pre-selected level, something not possible with traditional compound figures. Here, we used ***level specific letter presentation*** within the Hillyard paradigm. This not only allowed us to isolate the ERPs related to the two levels of the stimuli, it also permitted us to directly observe effects of attention specifically related to each level without contamination from the effects at the other level.

Two separate oddball sequences were presented at fast rates, one consisting of global letters and the other consisting of local letters. A set of four letter identities comprised the frequent distracter set, whereas two letter identities were included in the oddball target set which were the only stimuli for which responses were required. Previous work had demonstrated that it is difficult to divide attention between the two streams, especially when presentation rates are fast (Valdes-Sosa et al., 2014a). Note that the fast stimulation rates in the Hillyard paradigm, necessary to induce stringent attention selection, can have some undesirable side-effects. One of these is that although jittering the stimulus onsets serves to attenuate contamination of the ERP associated with one stimulus caused by overlapping responses to other stimuli, some pollution always survives. Since jittering acts as a low–pass filter, the problem is more severe for slow components triggered by previous events that intrude into the epochs of interest.

The ADJAR method (Woldorff, 1993) was developed to reduce this contamination, by applying an iterative de-convolution procedure in the time domain. However, ADJAR has proven relatively difficult to implement and is computationally intensive. Moreover it fails to remove overlap from some low-frequency responses (for an evaluation see Talsma and Woldorff, 2005). A more straightforward solution to this problem is to deconvolve the ERP responses associated to different stimuli by using regression within the general linear model (GLM) framework. This approach was used originally for functional magnetic resonance imaging (Poldrack et al., 2011) but has recently been extended to the skin conductance responses (Bach and Friston, 2013) and ERPs (Bardy et al., 2014; Smith and Kutas, 2014). It effectively solves the response overlap problem, and is the method used in the present article (see Methods section for our implementation of this approach).

The goal of this article was to compare the ERPs elicited by letters obtained when attention was focused on their channel of presentation (global or local) with the ERPs obtained when attention was diverted to the other channel. In a first experiment we verify the degree of attentional selection induced by our stimuli. We compared identification of targets when only one level was reported (focused attention) with recognition of targets when both levels had to be reported (divided attention). The results confirmed that performance was much worse when subjects attempted to attend both levels at the same time than when they focused on only one level. In the second and main experiment, we were able to establish that attention to one level enhanced a SN in the ERPs associated to events within that level. This was possible only because we were able to estimate separate ERPs for the global and local stimuli, in contrast with previous work in this field. Furthermore, attention at later latencies also enhanced the DN elicited by targets compared to distracters within the attended (but not the unattended) channel. A preliminary analysis of this data was presented in Valdes-Sosa et al. (2014a). These results are the first reports (to our knowledge) of attentional effects that can be unambiguously ascribed respectively to processing within the global and the local channels.

## Experiment 1

The goal of this article was to study the attentional effects on ERPs selectively associated to global and local channels with our novel stimuli. A pre-requisite to achieve this aim is that the defined channels compete vigorously for attention, a provision that was tested in this first experiment. Participants either focused attention at one level during some blocks of events, or tried to divide attention between the two levels in other blocks. If attention is difficult to share between levels, then more mistakes in letter identification should appear in the divided–than in the focused-attention blocks. Perceptual load and other factors such as memory and response demands (henceforth processing load) constituted a possible confound in the present experiment, since in the divided attention task the subject had to monitor twice as many targets as in the focused attention blocks. To control this, we also included focused attention blocks in which twice the number of targets were presented in each oddball stream of stimuli. We hypothesized that, if processing load is important for discriminating the targets, then the subject's performance should be poorer in these high-load blocks than in the low-load focused attention blocks.

### Methods

#### Participants

A group of five (two females) university graduate students from the Cuban Center for Neuroscience with ages between 25 and 35 were recruited. All subjects had normal, or corrected to normal vision, and none of them had a history of neuropsychiatric disorders, or were taking psychotropic drugs, at the time of this experiment. A written informed consent was obtained from all participants and the experimental protocol was approved by the ethics committee of the Cuban Center for Neuroscience.

#### Stimuli and titration of letter durations

Participants were presented with an array of “eight” characters, consisting of three columns and five rows, which served as a baseline and mask (Figure 1B). This array was presented at the center of a CRT screen, placed 40 cm in front of the observers. Light-gray characters (on a black background) were used. To reveal global stimuli (see Figure 1B for an example), a subset of complete “eight” characters was erased. For unveiling of the local letters (Figure 1B), a subset of the line segments making up the “eight” characters was erased. Therefore, symbols were still present at all positions. For these local letters, only 10 of the “eight” characters were changed in each trial (randomly selected out of the 15 possible). This was aimed at discouraging subjects from focusing attention on a few positions across trials.

At both levels, the erasures could create one hand two letters: “E,” “P,” which were designated as targets; and on the other hand four letters “H,” “U,” “S,” and “b” which were designated as distracters. Note that at any instant only the background mask, an isolated global letter, or an isolated local letter was displayed. Both types of letter never co-existed. Global letters were 100 mm high and 38 mm wide (approximately 7.2 × 2.43 degrees of visual angle). Local letters were 18 mm high and 10 mm wide (approximately 1.42 × 0.8 degrees of visual angle) (see Supplementary Materials for details).

Here, the stimulus duration of global and the local letters was titrated in each subject a separate session (before the main experiment) with a Quest staircase (implemented on Matlab 6.5, Mathworks Inc., see Watson and Pelli, 1983). Each trial consisted of a randomly selected letter presented for a time controlled by Quest, preceded and followed by a mask, both of which lasted 300 ms. The initial Quest parameters were: beta = 3.5, delta = 0.01, gamma = 0.5 and grain = 0.01, and titration was terminated when 80% correct recognition was achieved.

#### Procedure

In both experiments in this study, the stimulation protocol was controlled by using the COGENT toolbox (http://www.vislab.ucl.ac.uk/cogent.php) on Matlab 6.5 (Mathworks Inc.). Five blocks were presented each with 300 stimuli. In each block 60 target letters and 240 distracter letters were presented, each separated from its temporal neighbors by baseline intervals ranging between 600 and 800 ms, evenly distributed between levels. In one block, participants were presented with, and required to attend to, letters in both levels (Divided-Attention block). The same type of sequence was presented in two additional blocks in which participants were instructed to attend to only one level (Focused-Global, Focused-Local), thus reporting only 30 targets. Note that the target processing load was twice as large in the divided- than in the focused–attention blocks, which could in itself contribute to higher error rates. To control for this possible confound, additional focused attention blocks were included in which the 300 letters were presented only at the attended level, thus having to report 60 targets as in the divided attention situation (High-Load-Focused-Global and High-Load-Focused-Local). The global and local letter durations were determined by the titration procedure described above.

Participants were instructed to report the target letters at the level(s) designated for attention in the same sequence they were observed by pressing pre-designated keys, while ignoring all distracter letters as well as targets at the non-attended level. The Levenshtein distance (LD; Levenshtein, 1965; Gusfield, 1997) between the sequences of presented and reported target letters was calculated for each block (this distance is the minimum number of transformations–insertions/removals/replacements–needed to convert one sequence of characters into another, therefore indexing the mistakes made in reproducing the targets). The lower the distance the more similar the strings (i.e., LD = 0 implies that both string are identical). The maximum possible distance is equivalent to the maximum string length. After the titration procedure, the presentation time with the LD closer to 80% performance was selected as stimulus duration for the corresponding letter level. All these distances were submitted to a repeated measures ANOVA, using one within-subject factor: Condition (Divided-Attention vs. High-Load-Focused-Global vs. High-Load-Focused-Local vs. Focused-Global vs. Focused-Local). The Greenhouse-Geisser correction was used when appropriate (Greenhouse and Geisser, 1959). An additional ANOVA was performed on the focused-attention blocks with Attended-level (local vs. global) and Processing–load (high vs. low) as within-subject factors.

### Results and discussion

**Figure 2:**
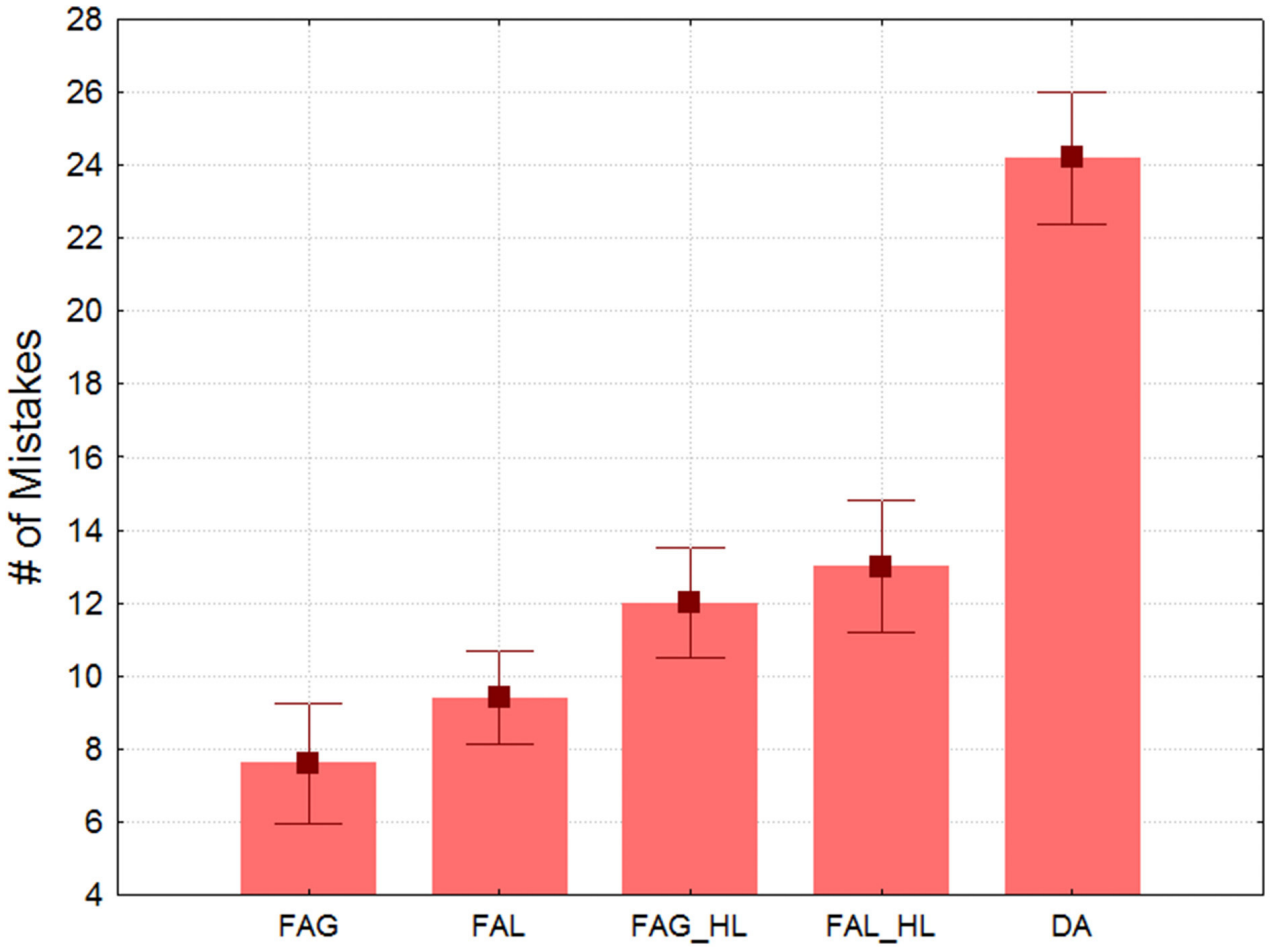
**Mean number of mistakes in target detection indexed by Levenshtein distance as a function of the block type**. Note the performance cost derived from dividing attention between both hierarchical levels. Whiskers indicate standard errors. FAG, low-load focused attention to global stimuli; FAL, low-load focused attention to local stimuli; FAG_HL, high-load focused attention to global stimuli; FAL_HL, high-load focused attention to local stimuli; DA, divided attention between global and local stimuli.

Figure 2 shows the mean amount of mistakes in each type of block across participants as indexed by the LD (remember larger LD values correspond to poorer performance). The main effect of Condition was highly significant [*F*_(4, 16)_ = 34.68, ε = 0.4318, *p* < 0.001]. In the ANOVA for focused attention blocks, the effect of Attended-level was not significant [*F*_(1, 4)_ = 4.7, n.s.], which was also true for Processing–load [*F*_(1, 4)_ = 9.4, *p* < 0.037]. The factors did not interact. However, planned comparisons showed that more mistakes were made in the divided attention trials than for the two high-load conditions [*F*_(1, 4)_ = 90.4, *p* < 0.001], an effect that was very strong. Therefore, the load confound cannot explain by itself the divided attention costs. Divided attention produces an interference that goes above and beyond the effect of processing load.

In this experiment we confirmed that identification and report of targets in the oddball sequence was significantly impaired by dividing attention between the two (global and local) concurrent oddball streams. This replicates results described in Valdes-Sosa et al. (2014a), but with the exact stimuli used in the Experiment 2 of the present article, and with a control for a processing load confound. The divided attention cost probably reflects sluggish attentional shifts between different the channels from different levels. Previous work with our stimuli show that in an attentional dwell-time design (Duncan et al., 1994) it is difficult to report the second of two successive targets letters when they belong to different levels if they are close together in time. In contrast, there is no interference for targets at the same level (Lopez et al., 2002; Valdes-Sosa et al., 2014a,b). This last effect probably explains the higher accuracy in the focused attention condition. These results indicate that our paradigm forces subjects to use a stringent attentional selection as described for other work using the Hillyard sustained attention (e.g., Proverbio and Mangun, 1994; Mangun and Buck, 1998).

## Experiment 2

In the Hillyard sustained attention paradigm, a stream of frequent distracters mixed with infrequent target stimuli is presented to one sensory channel (an “oddball sequence”). At the same time, a second stream is presented to another channel (the channels are usually locations in visual space or the two ears). Subjects have to report the targets in one stream. The timing of events in the two streams is asynchronous, enabling estimation of separate ERPs for all stimuli, and the pace is fast to impede effective division of attention between channels. Thus, attention can be directed to stimuli in one channel and then drawn away, allowing the effects of these maneuvers on the ERPs elicited within the same sensory channel to be directly compared. In this study, we considered each hierarchical level to be a channel, the separation of which we ensure with level specific letter presentation. Therefore, we presented subjects with several blocks consisting each of two randomly interspersed oddball streams, one global and one local. Attention was directed to one level in half of the blocks and to the other level in the other half.

### Methods

#### Participants

Initially, 15 healthy volunteers participated in our study. Two of them were discarded due to excessive noise in the EEG recordings and ocular movements. The remaining 13 participants (five females) had ages ranging from 23 to 30 years (mean = 29.5). All of them were right-handed by self-report, had normal, or corrected-to-normal vision, and none of them had a history of neuropsychiatric disorders, or were taking psychotropic drugs. All gave written informed consent prior to the experiment. The protocol was approved by the Ethics Committee of the Cuban Center for Neuroscience and was carried out according to the principles laid down in the Helsinki declaration.

#### Stimuli and procedure

The stimuli and paradigm used here was identical to that of the low-load focused attention blocks in Experiment 1. As in Experiment 1, each subject first participated in a separate session for titration of the optimal stimulus duration at each letter level. The means and standard deviations values of these durations were 59 ms (±24 ms) for global stimuli and 154 ms (±32 ms) for local stimuli. Four blocks stimulation blocks presented. In each block 480 letters were presented, each separated from its temporal neighbors by baseline intervals ranging from 600 to 800 ms. The blocks contained 180 global distracters, 180 local distracters, 60 global targets and 60 local targets. The order of letter presentation in each sequence was randomized. In two of the four blocks, subjects were instructed to attend only to the global stimuli (Attend-Global blocks), and in the other two they were instructed to attend only to the local targets (Attend-Local blocks). In seven participants, the order of presentation was global→local→global→local. In the other six participants the order was reversed. Subjects were required to report targets at the designated level by pressing one of two keys on a computer keyboard. Target (and distracter letters) in the non-attended level had to be ignored. The LD was calculated for each block.

#### Electrophysiological recordings

Electrophysiological recordings were performed with a MEDICID 64 system (Havana, NEURONIC SA.). Electrodes were placed in 58 active derivations referenced to the nose. In addition, four electrodes were used for recording the electro-oculogram: two lateral to the external border of each eye for horizontal movements, and two placed 1 cm above and below the left eye for vertical movements. The signal was amplified by a factor of 1000, and band-pass filters filtered between 0.05 and 70 Hz (−3 dB down). A/D conversion was performed at 200 Hz. Data segments of 800 ms were defined for analysis, starting 100 ms before each stimulus. Each segment was visually inspected and those presenting artifacts or excessive activity in EOG were rejected. The within subject averages for each distracter condition included on the average about 190 repetitions, and those corresponding to the target about 40.

#### Deconvolution method

The ERP epochs for each stimulus had a large degree of overlap from responses to previous trials due to the fast-paced design employed here. This contamination is accompanied by unstable pre-stimulus baselines. We therefore estimated the average ERPs of each condition in all individuals with a novel methodology (Trujillo-Barreto, submitted) dubbed “form-free un-mixing for ERPs” (FUN for ERPs). This is similar in purpose to the previously described ADJAR method (Woldorff, 1993). The logic underlying FUN, inspired by methods developed for fMRI analysis is described below.

In fMRI deconvolution is accomplished by applying the GLM. A design matrix is built in which each column corresponds to the convolution of a set of finite impulse response (FIR) functions with a time series containing the onsets of one stimulus type. The GLM model assumes that.

(1) Y=Xβ+∈

where *Y* is the (ntx1) vector of fMRI raw data (nt = number of time points), *X* is the (nt × nest^*^T) design matrix (nest = number of different stimuli and T = number of time samples of the HRF of each stimulus), is the amplitude or weight vector (nest^*^T × 1) corresponding to each stimulus, and ε is a column vector of Gaussian white noise terms (Worsley and Friston, 1995; Smith et al., 2007). A Least Square estimate of the amplitudes is obtained as follows:

(2) $$\hat{\beta }=\;{\left(X^{T}X\right)}^{-1}X^{T}Y$$

This approach deals effectively with the response overlap even in fast event-related fMRI designs where the inter-stimulus intervals are smaller than the duration of the hemodynamic response.

FUN consists of an adaptation of this approach to ERP responses. It uses a similar GLM model of the observed data in which the total ERP response at a given time point is expressed as a superposition of sets (blocks) of FIR filters, each set modeling a condition-specific response. FUN for ERPs implements a Variational Bayes (VB) inversion of this generative model under a Mean Field Approximation (MFA) that exploits the standard independency assumption between different condition-specific ERPs. Particularly, the MFA used assumes that the posterior distribution of the FIR coefficients factorizes over experimental conditions. That is, the FIR coefficients corresponding to different condition-specific ERPs are assumed to be independent given the observed data. Under this MFA, the FUN for ERPs method produces posterior estimates of the FIR coefficients based on which the ERP corresponding to each experimental condition can be reconstructed. Additionally, since the generative model underlying FUN for ERPs is expressed in the form of a GLM, it allows for including additional regressors accounting for different types of trends and artifacts. The method also allows for accommodating available prior information (temporal smoothness for example) about the ERPs. See Smith and Kutas (2014), for a different but related approach.

The mean of the pre-stimulus amplitudes was subtracted from each individual average waveform. After the average ERPs were estimated for all subjects, grand averages for the group were calculated for each condition. Difference waveforms were calculated by subtracting the waveforms generated by attended and unattended distracters for each hierarchical level, yielding selection negativity (SN), and by subtracting those elicited by attended targets and distracters, obtaining a DN.

#### Statistical analysis

Non-parametric permutation tests (Good, 2000) were used for the statistical tests with the ERP data given their non-Gaussian distribution and small sample sizes. Details of these tests can be found in Galán et al. (1997) and Maris and Oostenveld (2007), of which a brief review follows. Permutation tests were used in three situations: (1) comparisons between ERP time-series waveforms; (2) comparisons between summary measures extracted from these waveforms; and (3) comparisons between scalp distributions at selected time points.

***Comparisons between ERP time-series waveforms from different experimental conditions***. The subjects' waveforms for two conditions were compared with a paired *t*-test at each time point for all channels, first with the original condition labels and then after 1000 random permutations of the group membership labels. The significance (p) of the test under the null hypothesis (i.e., labels are exchangeable because conditions do not differ) is the proportion of *t*-tests for the permuted data that were greater than or equal to the original *t*-test. All contiguous time points with *p*-values < 0.05 were grouped into clusters, and the cluster-mass index was defined as the sum of the corresponding *t*-values. The maximum cluster-mass index across all derivations was used to build an empirical null distribution. This procedure controls the overall Type I error due to multiple comparisons (Maris and Oostenveld, 2007). The significance of the cluster-mass values from the original (un-permuted) data was then estimated from this empirical distribution. This procedure was applied to compare ERPs for attended/unattended distracters, for targets/distracters, as well as for homologous pairs of left/right electrodes for each condition.

***Comparisons between measures extracted from the ERP waveforms***. The latencies of both SN and DN for each hierarchical level were estimated as the time point at which the component reached 50% of its total area (as proposed by Luck, 1998, 2005), in the average of all the posterior electrodes. An estimate of SN and DN amplitude was obtained by extracting the amplitude at this time point. As a lateralization test, the average of the voltage values corresponding to the above-mentioned latency for every difference waveform was calculated for a group of occipito-temporal electrodes separately for the left (T7, C5, C3, TP7, CP5, CP3, P7, P5, P3, P1, PO5, and PO3) and the right side (T8, C6, C4, TP8, CP6, CP4, P8, P6, P4, P2, PO6, and PO4) of the scalp. All pair-wise comparisons across the four difference waveforms were tested by using the same permutation test described without cluster-based correction.

***Comparisons between scalp distributions at selected time points***. Variations in the scalp distributions of the SN and DN between the attend local and attend global waveforms were tested using the D statistic (Galán et al., 1997), which is based on the order of the electrodes when ranked by amplitude (this eliminates the amplitude/distribution confound discussed by McCarthy and Wood (1985). The D statistic is the sum of the Euclidean distances between the positions of the pairs of electrodes that have the same rank across the two conditions. The significance of D is obtained by comparing its value for the original observations with an empirical distribution of obtained after 1000 random permutations of the group membership labels (under the null hypothesis that the groups are equivalent). This permutation test is distribution free, making no assumptions about the underlying correlation structure.

### Results and discussion

#### Behavioral data

Task performance was accurate in all subjects. The mean LD for global stimuli was about 19.92 (about 83.4% accuracy). The mean LD for local stimuli was about 25.38 (about 78.8% accuracy). The difference in LD between global and local stimuli was not significant [*t*_(12)_ = 1.93, *p* = 0.077].

#### Global ERPs

**Figure 3:**
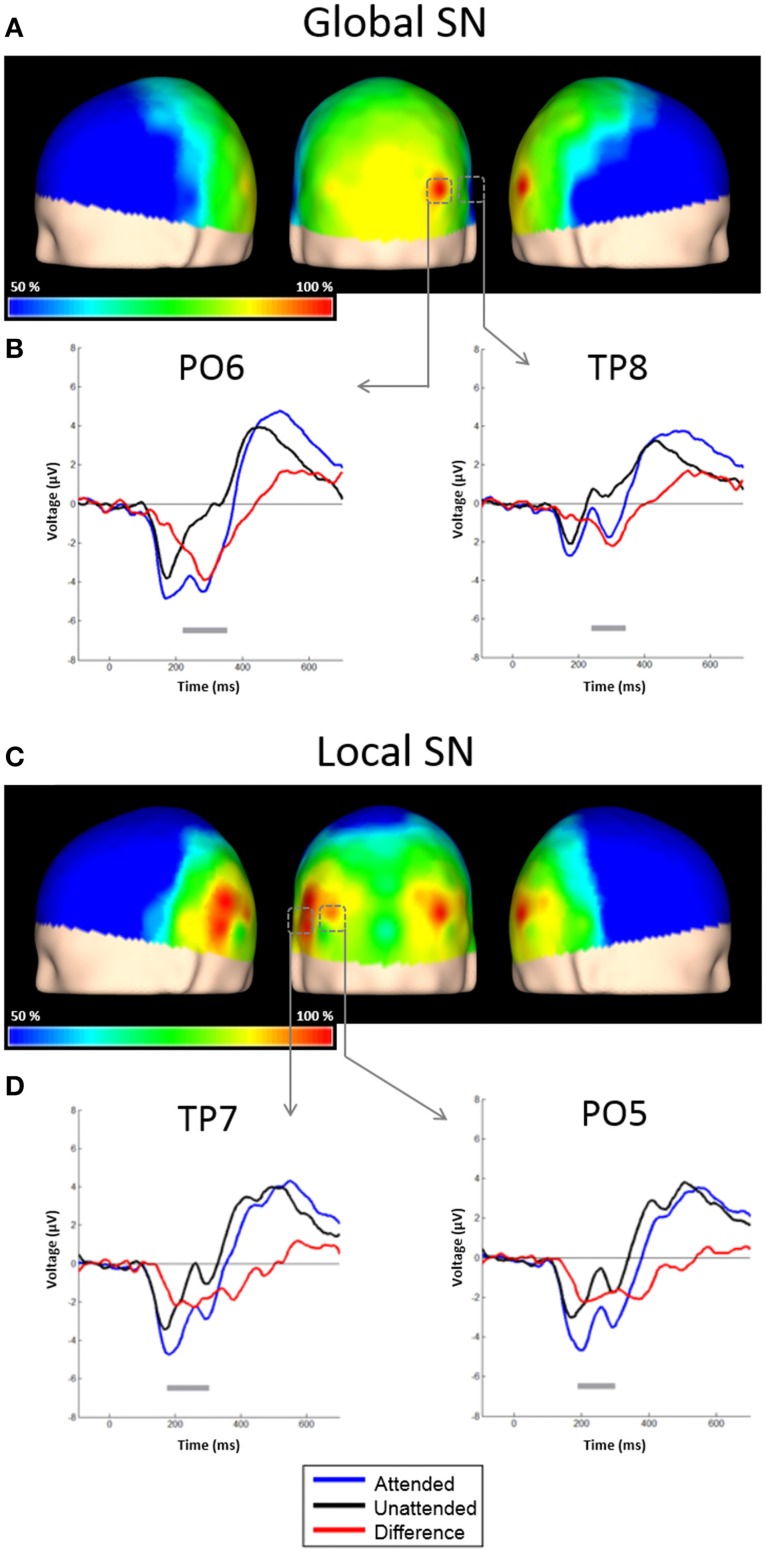
**Scalp topographies of the SN and representative examples of the grand average ERPs to distracters. (A)** Scalp distribution of the SN obtained by subtracting ERPs to unattended global distracters from ERPs to attended global distracters, and interpolating the amplitudes measured at all electrodes. Results are plotted as the percentage of the maximum value, at the 50% area latency. **(B)** ERPs from derivations PO6 and TP8. Responses to attended global stimuli (blue solid line), unattended (black solid line) and the difference waveform (red solid line) are overlaid. Significant effects (largest *t*-test values) were found in these electrodes. Gray horizontal lines indicate latencies with significant differences between attended and un-attended conditions (*p* < 0.05, corrected). **(C)** Scalp distribution of the SN obtained by subtracting ERPs to unattended global distracters from ERPs to attended global distracters, and interpolating the amplitudes measured at all electrodes. Conventions here as in **(A)**. **(D)** ERPs from derivations PO5 and TP7. Conventions here as in **(B)**.

Success in de-convolving the responses to different stimuli (and the elimination of baseline contamination) can be assessed by examination of the ERP pre-stimulus time windows. In all the individual cases, as well as the group averages, the waveforms in these time windows were flat, indicating a successful de-convolution. The ERPs associated with global distracters were characterized by several large peaks over the occipito-temporal regions of the scalp. As shown in Figure 3B, there were two large negative peaks in posterior areas for both attentional conditions, with 50% area latencies of about 175 and 285 ms after stimuli onset respectively (N1 and N2). These peaks were followed by a positive peak, also larger over posterior regions, with a latency of about 525 ms.

Attentional effects for global distracters were observed in the time window occupied by the N1 and N2 components, where the ERPs were more negative for the attended condition. A broad negativity, spanning part of N1, a small positive peak, and N2, seems responsible for this effect. In other words, this is not a simple modulation of the amplitude of the exogenous N1 and N2, but instead seems to be the superposition of an endogenous negativity on these peaks. We will dub this negativity as SN. The effect was significant in 30 out of 58 active derivations, and was concentrated in posterior occipital, temporal and parietal derivations. In all these electrodes, the *p*-values of the cluster-mass corrected permutation tests were consistently below 0.0001. The largest effects were found in derivations PO6 and TP8, on the right side of the scalp (Figure 3A). In these electrodes, the difference between attended and un-attended waveforms was significant for latencies ranging from about 220 to 350 ms. Interestingly, the global SN was lateralized to the right hemisphere. The permutation test using the average amplitude across posterior occipito-temporal electrodes, showed that this hemispheric asymmetry was significant (*p* = 0.002) (**Figure 6**, left panel).

**Figure 4:**
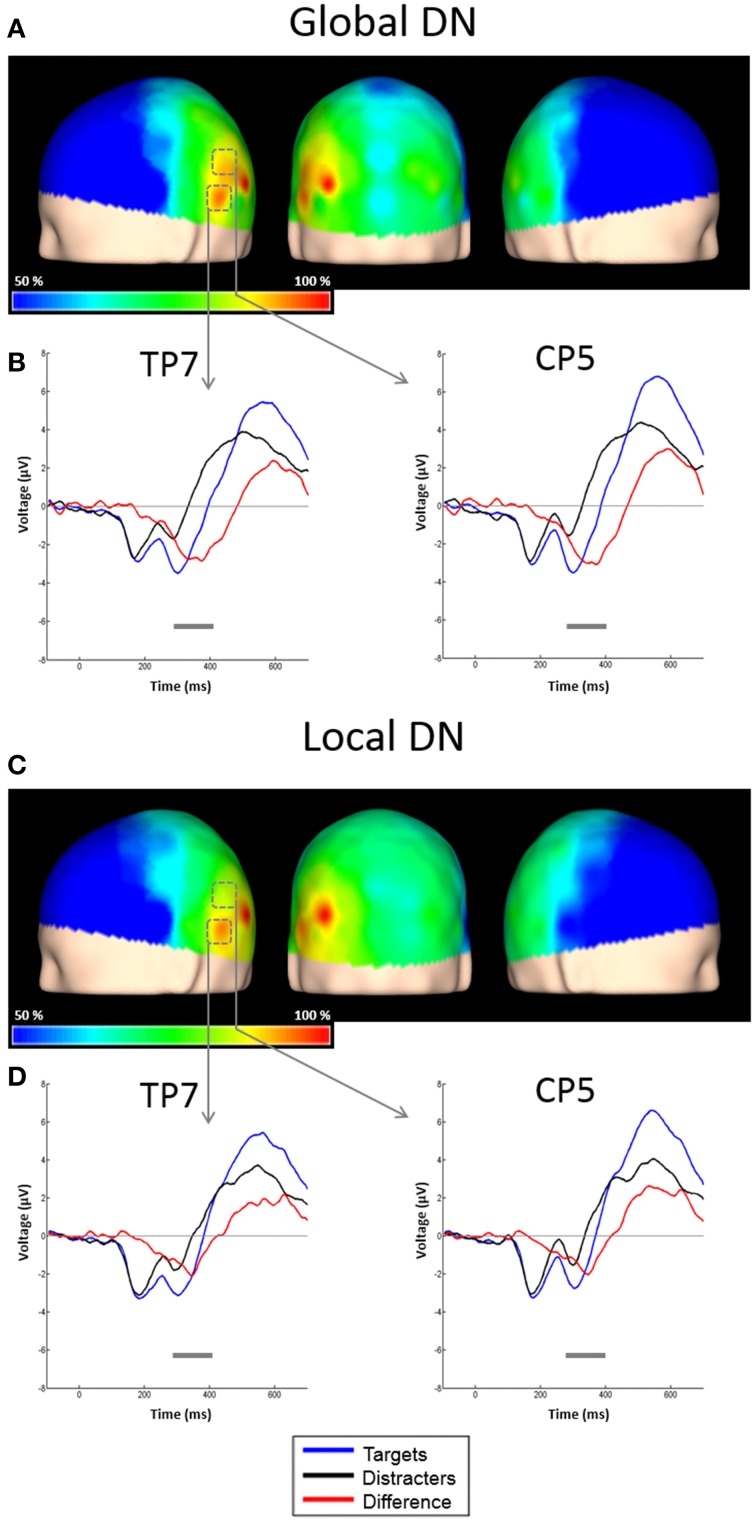
**Scalp topographies of the DN and representative examples of the grand average ERPs to targets and distracters. (A)** Scalp distribution of the DN obtained by subtracting ERPs to attended global distracters from ERPs to attended global targets, and interpolating the amplitudes measured at all electrodes. Results are plotted as the percentage of the maximum value, at the 50% area latency. **(B)** ERPs from derivations CP5 and TP7. Responses to attended global targets (blue solid line), attended global distracters (black solid line) and the difference waveform (red solid line) are overlaid. Significant effects (largest *t*-test values) were found in these electrodes. Gray horizontal lines indicate latencies with significant differences between attended targets and distracters (*p* < 0.05, corrected). **(C)** Scalp distribution of the DN obtained by subtracting ERPs to attended global distracters from ERPs to attended global targets, and interpolating the amplitudes measured at all electrodes. Conventions here as in **(A)**. **(D)** ERPs from derivations CP5 and TP7. Conventions here as in **(B)**.

**Figure 5:**
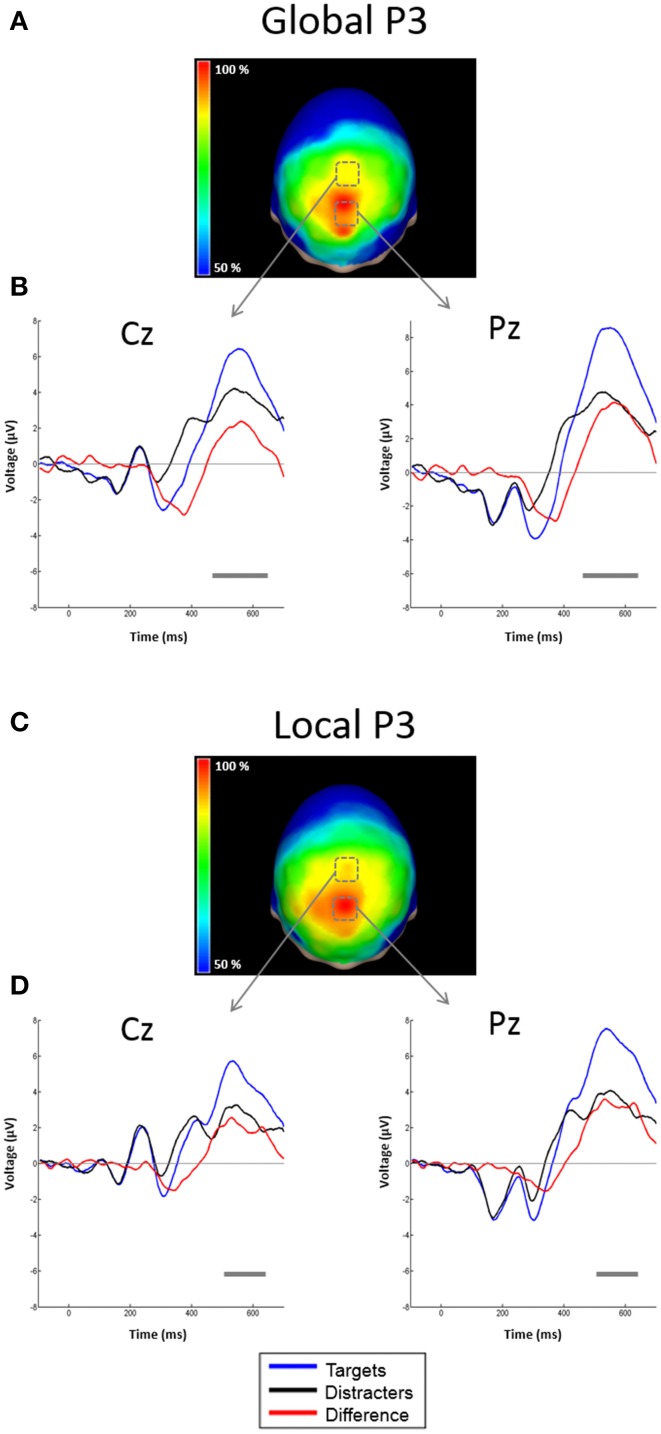
**Scalp topographies of the P3 and representative examples of the grand average ERPs to targets and distracters. (A)** Scalp distribution of the P3 obtained by subtracting ERPs to attended global distracters from ERPs to attended global targets. Conventions here as in Figure 4, but measured at P3 50% area latency. **(B)** ERPs from derivations Cz and Pz. Conventions here as in Figure 4. **(C)** Scalp distribution of the P3 obtained by subtracting ERPs to attended local distracters from ERPs to attended local targets. Conventions here as in Figure 4, but measured at P3 50% area latency. **(D)** ERPs from derivations Cz and Pz. Conventions here as in Figure 4.

**Figure 6:**
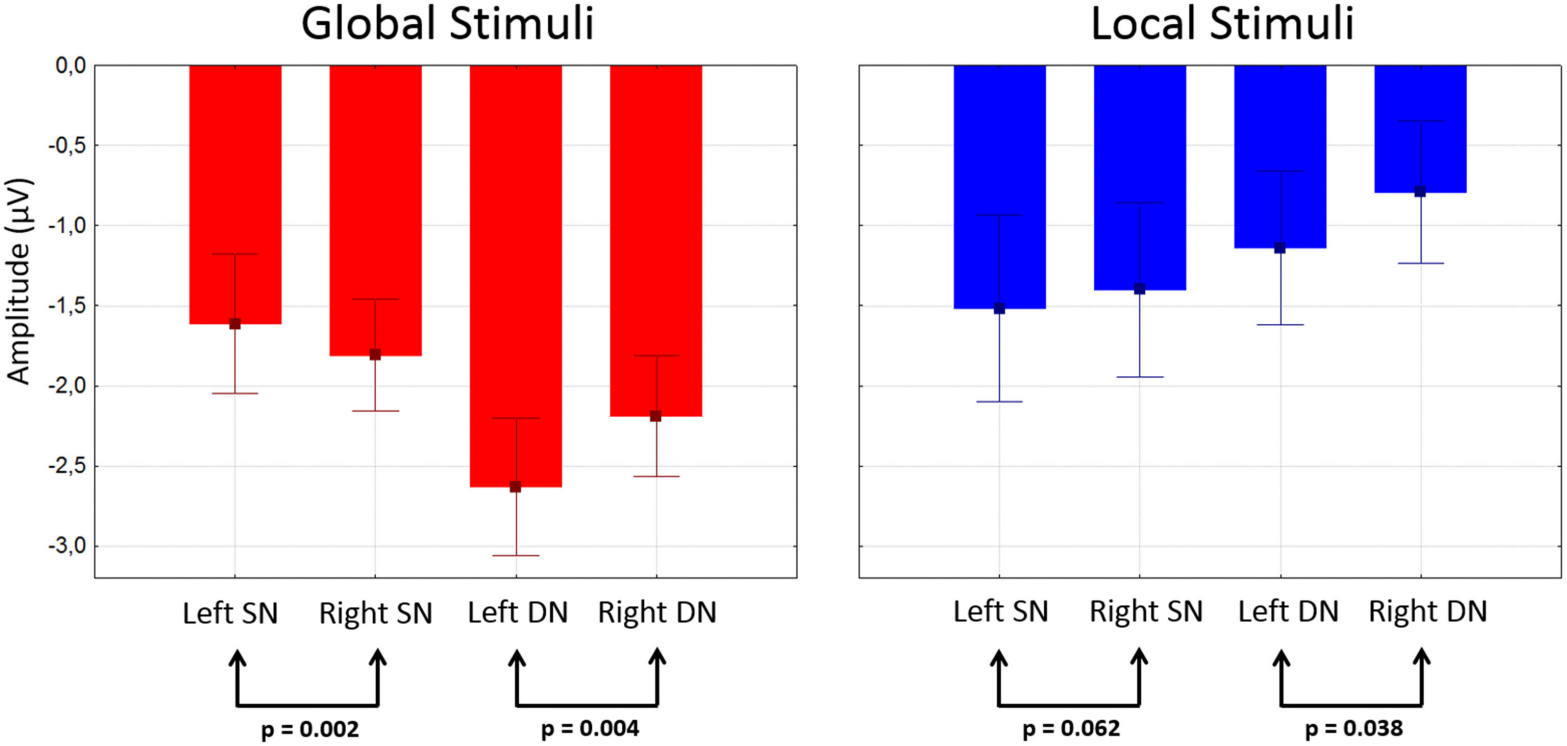
**Mean amplitudes of SN and DN at posterior electrodes as a function of stimulus type and hemisphere**. Amplitudes were measured in the corresponding difference waveform at the 50% area latency for each subject after averaging data from posterior electrodes in each hemisphere (see Methods). Whiskers indicate standard errors.

The ERPs to the unattended stimuli did not differ statistically between targets and distracters at any time point across all electrodes. A comparison of responses to attended targets and distracters, separately for each level, is shown in Figure 4. Attended targets produced a large N2c/P3b complex that was absent in the responses to distracters. The P3b was largest at central and posterior derivations (Figures 5A,B). For global stimuli this component spanned from 455 to 700 ms. Subtraction of the waveforms elicited by attended targets and distracters allowed us to measure the DN (corresponding to oddball triggered N2c). This effect was significant 18 out of 58 active derivations, and was concentrated mainly in left temporal and parietal derivations (Figure 4A). The largest effects were found in derivations TP7 and CP5, on the left side of the scalp (Figure 4B). In these electrodes, the difference between waveforms elicited by attended targets and distracters was significant for latencies ranging from about 275–420 ms. The global DN was strongly lateralized to the left hemisphere (*p* = 0.004 see Figure 4A and left panel of Figure 6).

#### Local ERPs

Figure 4 shows the ERPs elicited by local distracters. These were very similar to the waveforms for global stimuli. N1 and N2 50% area latencies were about 175 and 300 ms respectively (Figure 4). A late positive component peaking at about 505 ms was also observed. In this case, ERPs triggered by attended distracters also produced an enhanced negativity (SN) compared to ERPs for the unattended distracters. As for the global distracters, this effect is not a modulation of N1 or N2, but a superimposed component that spans several peaks. The difference between these responses was significant in the permutation tests for 11 out of 58 active derivations, concentrated in posterior occipital and temporal derivations. In all these electrodes, the smallest *p*-values were all below 0.001 (cluster-mass corrected). The largest effects were found in derivations PO5 and TP7, on the left side of the scalp (Figures 3C,D). In these electrodes the significant difference between attended and un-attended waveforms started and ended at about 185 and 300 ms in that order. Although the local SN showed a tendency to be lateralized to the left hemisphere, this component was more bilateral than the SN for global distracters and thus only marginally significant (*p* = 0.062, Figure 3C and right panel of Figure 6).

Similar to global stimuli, the ERPs elicited by unattended stimuli did not differ statistically for targets and distracters at any time point across all electrodes. Attended targets also produced a large N2c/P3b complex that was absent in the responses to distracters. The P3b was largest at central and posterior derivations, spanning from 480 to 700 ms (Figures 5C,D). The DN waveform was present in 20 out of 58 active derivations, and it was also concentrated mainly in left temporal and parietal derivations (Figure 4C). The largest effects were found in derivations TP7 and CP5, on the left side of the scalp. In these electrodes the difference between waveforms elicited by attended targets and distracters was significant for latencies ranging from about 295–365 ms (Figure 4D). The local DNs was lateralized to the left hemisphere (*p* = 0.038, see Figure 4C and right panel of Figure 6).

#### Comparisons between conditions and components

**Figure 7:**
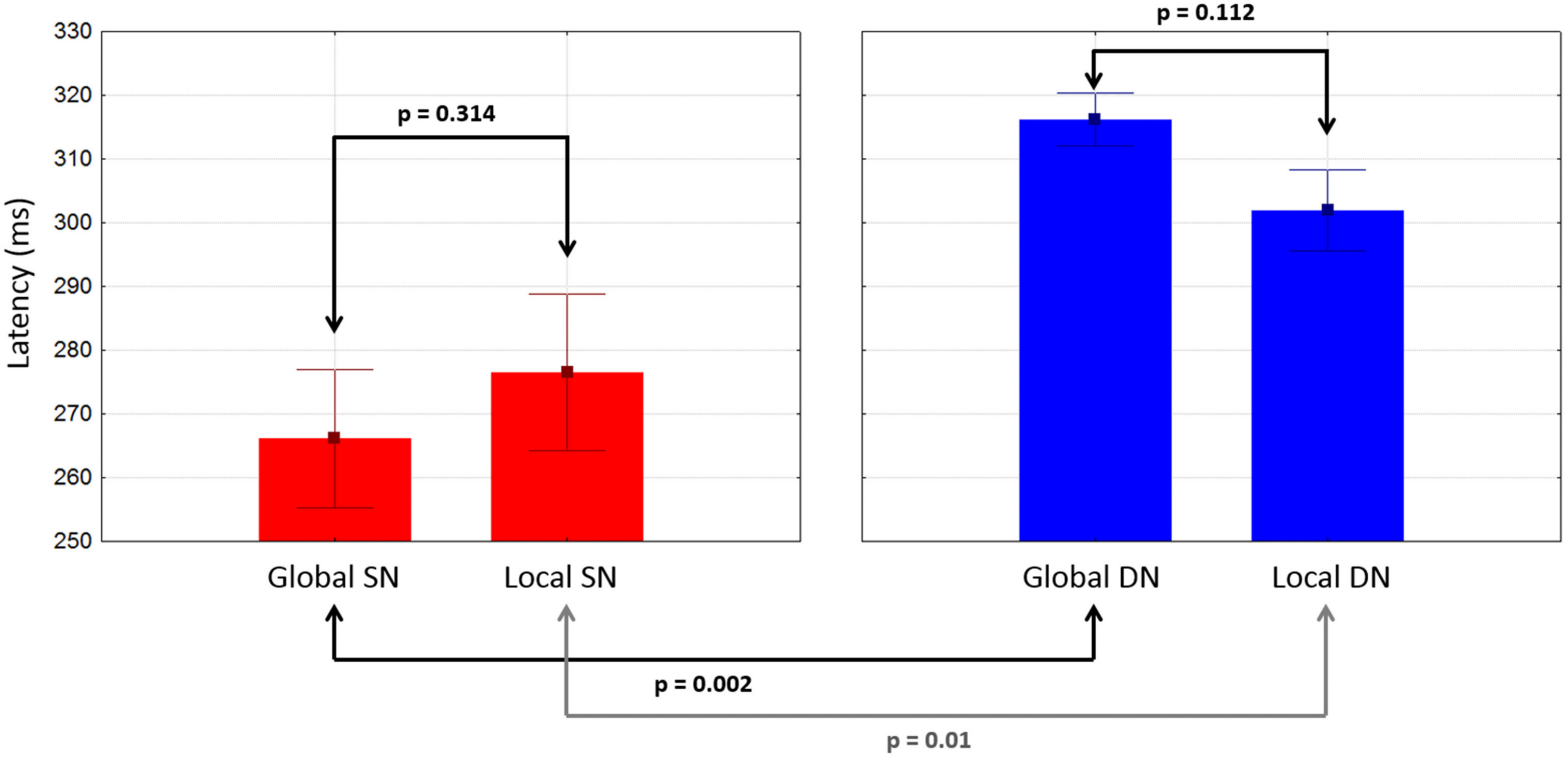
**Mean latencies of SN and DN at posterior electrodes as a function of stimulus type**. Whiskers indicate standard errors.

There were no significant differences between global and local ERP amplitudes within each attention condition. The 50% area latencies of the SN did not differ between global and local distracters (*p* = 0.314) (Figure 7, left panel). Thus, the latency differences between the global and local levels found in our preliminary analysis (Valdes-Sosa et al., 2014a) were not confirmed. Interestingly, the global SN waveform was about 50 ms earlier than the global DN (*p* = 0.002) (Figure 7). Similarly, the local SN was about 25 ms earlier than the local DN (*p* = 0.01) (Figure 7). Comparison of the scalp topographies of the SN for global and local stimuli, (shown in Figures 3A,C) showed that although both exhibited a posterior distribution over occipito-temporal regions, their scalp topographies were significantly different (D statistics, *p* < 0.01). In contrast to SN, both the global and the local DNs were strongly lateralized to the left hemisphere (*p* = 0.004 and *p* = 0.038 respectively, see Figures 4A,C), and their scalp topographies did not differ (D statistics, *p* = 0.242). On the other hand significant differences in scalp distribution were found between the SN and DN waveforms for global (D statistics, *p* < 0.001), but not for local stimuli (D statistics, *p* = 0.084).

Note that earlier responses that can precede N1 (such as P1 and C1 were not present). The P1 has been found in previous ERP studies of compound figures (e.g., Heinze and Münte, 1993; Schatz and Erlandson, 2003). Perhaps these very early peaks were not found because our letters do not differ enough from the background in luminance, contrast, and number of line segments, as well as the fact that these letters were created by offsets of pixels instead of onsets. This may have reduced the C1 and P1 components which are very sensitive to the luminance, contrast and spatial frequency of the stimuli (Seiple et al., 2002; Proverbio et al., 2007). SF composition is known to affect C1 and P1 amplitudes, and helps differentiate ERPs triggered by stimuli with different SF content (Martínez et al., 2001; Flevaris et al., 2014). The absence these early components probably explains why ERPs to local and global letters did not differ in morphology despite their very different SF content (see Valdes-Sosa et al., 2014b for representative spectra). Further experiments with our same procedure, but modifying the stimuli so they can elicit a measureable C1 or P1, are needed to completely exclude global/local attentional effects in this time window. Robust SN and DN attentional effects were found for both global and local letters. Note that previous work (Han et al., 1997, 1999, 2001; Volberg and Hübner, 2004; Machinskaya et al., 2010) with global/local selection could not dissect these attentional effects that are specific to each level. The mandatory synchrony of local and global information onsets/offsets for the traditional compound figures used in that work, effectively precludes any attempt to obtain a deconvolution of the responses associated with the two levels. A more extended discussion of SN and DN is deferred to the general discussion. We finally observe that the independence of P3b scalp topography was expected since this component has been shown to reflect modality independent processes, such as the updating of working memory (Donchin, 1981; Donchin and Coles, 1988; Polich, 2007).

## General discussion

Using the combination of a novel paradigm, modified compound figures, and an original regression method for signal extraction, we were able to separate the neural responses triggered by letters at the global level from those triggered by letters at the local level. This allowed us to separately assess the effects of attention for each level, which is not possible with previously described methods. Using the Hillyard sustained attention paradigm, we first verified that accuracy of target recognition was severely impaired when attention was split across both local and global levels compared to when it was focused on only one level, an effect which was not due to the increased processing load of the former condition. Importantly, we found that the frequent distracter letters at the global level exhibited a posterior SN (larger over the right side of the scalp) when they were the focus of attention relative to when attention was diverted to the local level. Attended–relative to unattended–distracters at the local level elicited a more bilateral SN that was slightly larger over the left hemisphere. Recognized rare targets from an attended level produced an occipital-temporal DN, which was clearly larger over the left side for both global and local letters, and that was followed by a P3b.

The SN appears to be an endogenously generated component, similar to those present during the selection of non-spatial features such as color (Hillyard and Münte, 1984; Anllo-Vento et al., 1998), checkerboard size (Harter and Previc, 1978) and interestingly spatial frequency (Zani and Proverbio, 1997; Martínez et al., 2001; Baas et al., 2002). Selection of these non-spatial features also does not modulate the very early ERP peaks (but see Zani and Proverbio, 1995, 1997). SNs apparently index persistent processing of stimuli within the sensory channel defined by the relevant feature.

The SN scalp distribution varies moderately when different features are selected (Hillyard and Anllo-Vento, 1998; Martínez et al., 2001), which reflects partly distinct neural sources. The topography of our SNs are consistent with a greater involvement of the right hemisphere in processing of global letters, and a relatively larger involvement of left hemisphere in processing the local stimuli, although processing in this case may be more bilateral. This lateralization is consistent with psychophysical and neuropsychological evidence for hemispheric specialization of global/local processing (Heinze and Münte, 1993; Schatz and Erlandson, 2003; Volberg and Hübner, 2004; Flevaris et al., 2011). Since previous studies have not used our exact stimuli and procedures, direct comparisons between our results and pre-existing literature is not possible. However, some earlier work has yielded results with suggestive similarities to ours. For example, Martínez et al. (2001) presented high spatial frequency (HSF) and low spatial frequency (LSF) checkerboards as stimuli in the Hillyard sustained attention paradigm. The SN for the LSF stimuli was clearly larger over the right cerebral hemisphere, with a trend for larger SN over the left side for the HSF stimuli. Given that global information is thought to be carried by LSF bands whereas local information would be carried by HSF bands (Shulman et al., 1986; Shulman and Wilson, 1987; Robertson, 1996; Flevaris et al., 2011), the results of Martínez et al. (2001) are consistent with our findings.

Flevaris et al. (2014, this issue) have introduced an ERP design, that although very different from ours, also investigates attentional effects specifically related to the global or the local information. It takes advantage of a facilitation of perception for LSF/HSF gratings induced respectively by the previous recognition of global/local targets (Flevaris et al., 2011). Bilateral hierarchical letters were presented, on which LSF and HSF foveal grating probes were subsequently superimposed. The ERPs elicited by probes exhibited prominent P1 and P2 peaks (and also C1 for the HSF probe). P2 (but not C1 or P1) were larger for HSF probes on local targets (with a bilateral scalp distribution) and larger for LSF probes on global targets (larger over the right hemisphere). Although ignored, the probes benefited from the facilitation of the appropriate channel, with P1 lateralization as a function of level similar to that described here for the SN. Incidentally, SNs were not observed in this study, since the probes were irrelevant and unattended.

Space-based selection based on a “zoom-lens” are inconsistent with the ability to focus attention well by introducing a random placement for our local stimuli. However, sensory filtering by SF bands would be compatible with our results. This idea is supported by the pattern of scalp distributions for the SN as a function of level (larger over right for global, more bilateral for local). Congruently, the SN triggered by attention to LSF checkboards (Martínez et al., 2001), and the facilitation of P1 for LSF probes during attention to the global level of a hierarchical letter (Flevaris et al., 2014) are larger over the right hemisphere. Remember that LSFs are thought to carry global information. Moreover, the SN triggered by attention to HSF checkboards, and the facilitation of P1 for HSF probes when attending global letters letter, both have a more bilateral scalp distribution, albeit slightly larger over the left side. This also fits with the idea that HSFs carry local information. Flevaris et al. (2011) also obtained a similar pattern of results with EEG alpha-band suppression during preparation for targets at the corresponding level.

We also found a DN (about 30–50 ms tardier than SN) associated with the rare targets at both levels. Targets are known to trigger a N2/P3b complex in oddball paradigms. This N2 is thought to consist of several sub-components: N2a, N2b, and N2c (Pritchard et al., 1991). Specifically, N2c amplitude is larger for targets than for standard stimuli and its latency covaries with reaction time. Its scalp distribution is modality specific (Simson et al., 1976, 1977), being posterior (occipital-temporal) for visual stimuli but fronto-central for auditory stimuli. N2c seems to be equivalent to our DN. The N2c is thought to reflect a sub-process of stimulus classification (Folstein and Van Petten, 2008), since its only appears after successful recognition of attended targets.

The dependence of N2c topography on modality may extend to sub-modalities. In other words, its scalp distribution may differ for different selected features (Harter and Guido, 1980; Harter and Aine, 1984; O'Donnell et al., 1997) or categories of visual stimuli. For example, Harter and Aine (1984) found that the N2c was greater above the left hemisphere for selection of shape, color, feature conjunctions, and word discriminations, but not for spatial selection of visual stimuli. Therefore, N2c topography may reflect processing within diverse visual processing streams The predominantly left hemisphere distribution of our DN (equivalent to N2c) perhaps reflects the linguistic nature of the basic task (letter identification), which is independent of the level of target presentation. The invariance of DN topography across the two levels suggest that, in contrast to the SN, it is associated with a processing stage in which information about low-level physical details have been eschewed. Another component, the N2pc has been shown to be elicited during attentional selection, for example during visual search (see review by Luck, 2012). Its relationship to our SNs and DNs is not clear. The hallmark of this component is its lateralization on the scalp contra-lateral to the hemi-field of presentation of a target. Its amplitude is larger as a function of the number of distracters that need to be ignored or “filtered out.” Since our stimuli were always presented centrally, the critical test of N2pc presence could not be performed. However, our experiment could be repeated with bilateral stimuli in the two hemispheres pre-cueing the subjects toward one visual hemi-field.

It would be important to relate the two attention related components (SN and DN) found here to processing stages within theoretical models of global/local selection. There has been some debate on how early is global/local selection implemented. Several authors (Navon and Norman, 1983; Hughes et al., 1984) have suggested that this effect must be localized in very early stages of processing. However, other studies (Miller, 1981a,b; Boer and Keuss, 1982) have proposed a post-perceptual locus. More specifically, some models propose early sensory filtering based on SF bands which would in effect constitute the local and global channels (i.e., Flevaris et al., 2010), whereas posit spatial filtering (based on a “zoom-lens”) corresponding to the different sizes of global and local letters (Stoffer, 1993). In this special topic issue, Hübner proposes a dual-stage two-phase (DSTP) model (Hübner and Kruse, 2011; Hübner, 2014), with a first stage in which either the local or the global sensory channel receives greater attentional weighting (i.e., facilitation) as a function of the subjects goals or recent sensory history, which in turn determines an initial rate of evidence accumulation about the letters. A second (later and post-perceptual) stage contributes further to–selection via a content-to-level binding. The later stage is necessary to explain illusory conjunctions of letter identity and level (Hübner, 2014).

The SN is a possible index of sensory filtering in all models (including Hübner's initial stage). The difference in scalp topography for the SN between global and local stimuli suggests that this filter is implemented by partially distinct neural substrates for the two levels. On the other hand, the DN is a possible index of a second selection stage (perhaps the same as the second one proposed by Hübner). Its invariant scalp distribution suggests that is implemented by the same neural substrate for both levels. The onset latencies of the two components places an upper bound on the timing for the launching of the respective mental operations.

## Conclusions

The methods presented here successfully separate the ERPs elicited by the local and the global channels for hierarchically organized compound figures, and allow visualization of attentional effects for each level on its own. The paradigm effectively forced the subjects to focus attention on only one channel (local or global). We found two negative components related to attentional selection that partially overlapped in time. The first of these, a SN present for all stimuli in the attended channel and is probably mediated by different neural substrates for the local and the global level A DN with later onset, is triggered during by target recognition within the attended channel and has the same scalp distribution for both levels.

These negativities could respectively index two sequentially triggered (albeit temporarily overlapped) processing stages. A first process, reflected by the SN, could correspond to perceptual attentional filtering of stimuli based on the SF channels carrying each hierarchical levels. This process would differ in its mobilization of cortex across cerebral hemispheres. The second process, reflected by the DN, would reflect post-perceptual processes that arise after abstract letter form is distilled from the idiosyncratic low level formats associated to each hierarchical level. Its independence from the physical format of the letter would allow its implementation in a common neural substrate.

The segregation of neural responses and attentional effects for the global and local levels makes it possible to understand better the neural basis of hierarchical perception by disassembling the traditional compound figure. For example source localization methods, used identify neural generators of the ERPs, can be applied to the level-specific attentional effects, as has been done for the SNs obtained with other stimuli (e.g., attention to spatial frequency Martínez et al., 2001). Furthermore, separating the onset in time of global and local information enables not only more powerful ERP designs, but also pithier functional magnetic resonance imaging strategies, including the use of multivariate pattern analysis for which clearly segregated global and local exemplars are need to train classifiers. Classifiers to decode letters in ERP or fMRI data, with the global and local levels segregated (as possible with our stimuli), can then be used with traditional compound figures in which both levels are simultaneously present. This could offer a window into the competition between levels, the fate of the non-target level, and the origin of content-level mis-bindings.

### Conflict of interest statement

The authors declare that the research was conducted in the absence of any commercial or financial relationships that could be construed as a potential conflict of interest.
